# Unadjusted point of care creatinine results overestimate acute kidney injury incidence during field testing in Guatemala

**DOI:** 10.1371/journal.pone.0204614

**Published:** 2018-09-27

**Authors:** Benjamin R. Griffin, Jaime Butler-Dawson, Miranda Dally, Lyndsay Krisher, Alex Cruz, David Weitzenkamp, Cecilia Sorensen, Liliana Tenney, Richard J. Johnson, Lee S. Newman

**Affiliations:** 1 Division of Renal Diseases and Hypertension/Department of Medicine, University of Colorado Anschutz Medical Campus, Aurora, CO, United States of America; 2 Center for Health, Work & Environment, Colorado School of Public Health, University of Colorado Denver, Aurora, CO, United States of America; 3 Colorado Consortium on Climate Change and Human Health, University of Colorado Denver, Aurora, CO, United States of America; 4 Pantaleon, Guatemala City, Guatemala; 5 Department of Biostatistics and Informatics, Colorado School of Public Health, University of Colorado Denver, Aurora, CO, United States of America; 6 Department of Emergency Medicine, School of Medicine, University of Colorado Denver, Aurora, CO, United States of America; 7 Department of Environmental and Occupational Health, Colorado School of Public Health, University of Colorado Denver, Aurora, CO, United States of America; 8 Department of Epidemiology, Colorado School of Public Health, University of Colorado Denver, Aurora, CO, United States of America; International University of Health and Welfare, School of Medicine, JAPAN

## Abstract

**Objective:**

Acute kidney injury (AKI) occurs at high rates among agricultural workers (12–33%) in tropical environments. Because of the remote locations affected, traditional laboratory services are often unavailable. In this study we compare point of care (POC) creatinine values to standardized laboratory values, and examine the effect of POC testing on the interpretation of AKI rates under tropical field conditions.

**Methods:**

Blood samples were collected from 104 sugarcane workers from two time points in January 2018 as a derivation cohort, and from 105 workers from February to April 2017 as a validation cohort. Finger stick and venipuncture samples were drawn at the end of a worker’s shift to measure creatinine. Laboratory samples were tested in Guatemala City, Guatemala, in duplicate using the Jaffe Generation 2 method. An adjustment factor to improve agreement with serum creatinine was statistically derived and validated, and then used to determine impact on observed rates of acute kidney injury based on across shift changes in creatinine.

**Results:**

POC creatinine and serum creatinine measures showed that POC consistently overestimated the creatinine by an average of 22% (95% CI: 19.8%, 24.7%) and the disagreement appeared greater at higher values of serum creatinine. An adjustment factor of 0.7775 was applied, which led to significantly greater agreement between the two measures. Rates of AKI in the two combined groups fell from 72% before adjustment to 57% afterwards.

**Conclusions:**

POC testing under tropical field conditions routinely overestimates creatinine compared to laboratory testing, which leads to overestimation of rates of acute kidney injury. The application of an adjustment factor significantly improved the accuracy of the POC value.

## Introduction

Over the past two decades, a marked rise in the rates of kidney disease has been observed among agricultural communities worldwide[1], especially among workers in the sugar cane fields[2] along the Pacific Coast of Central America[3–6]. There have been an estimated 20,000 deaths in Central America attributed to the epidemic of “Mesoamerican Nephropathy”[7, 8], making it a major cause of mortality among an otherwise young and able-bodied demographic. While initial reports focused on rates of chronic kidney disease (CKD) in these populations[9, 10], more recent studies suggest agricultural workers are at high risk for recurrent episodes of acute kidney injury (AKI)[11, 12], which may contribute to the later development of CKD[13]. For example, Moyce et al. recently reported elevated rates of cumulative AKI among agricultural workers in California, with an 11.8% rate of AKI in a single day of summer field work[12]. A study by Mix et al. found that 81% of agricultural workers in Florida were dehydrated by the end of their shift as evidenced by specific gravity >1.020, and 33% of participants had at least one episode of AKI. Furthermore, the odds of AKI increased 47% for each 5-degree (°F) increase in the heat index[11]. The etiology of AKI in these populations has been an area of intense research, and possible explanations include exposure to toxins, chemicals, and heavy metals, infections, or effects from acute heat stress and dehydration[14–17].

To better understand the etiology of AKI and CKD in agricultural communities, researchers are increasingly conducting field studies comparing pre- and post-shift creatinine values. Similarly, agribusinesses, especially in the sugar cane industry, have begun to screen potential workers for reduced kidney function prior to the start of the harvest season. Because these agricultural communities are often found in remote or isolated areas, traditional laboratory studies are not readily available. A reliable point-of-care (POC) test is therefore critically important in making an accurate assessment of kidney function in these resource-limited settings.

Serum creatinine measurements using POC devices are generally considered to show acceptable agreement with gold standard laboratory measurement when used in emergency departments, intensive care units, and other healthcare settings in the United States[18, 19]. Dashevsky et al. recently reported a 94.5% level of agreement within 0.3 mg/dL[20]. However some studies have found variable concordance, especially in patients with worsening kidney function[21]. One potential reason for a discrepancy between POC and laboratory measures is that different methods of measurement are used. Creatinine in laboratory settings is often measured using a modified Jaffe method, while POC assays are often based on enzymatic or picric acid methods. These methods can give differences in results that have been shown to be clinically significant[22, 23]. In addition, POC assays can be more prone to interference than lab assays[24, 25].

The Statscan handheld device (StatSensor Xpress, Nova Biomedical, Waltham, MA, USA) is a POC device that utilizes an enzymatic method with amperometric biosensor[26], and has been utilized in field studies, including CKD screening in Nicaragua[9], and in AKI screening in California and Florida[11, 12] However, we are unaware of previous AKI studies that have examined reliability and validity of these instruments in more complex settings such as in international, tropical field conditions. We hypothesized that if POC creatinine measurements either under- or over-estimate serum creatinine, calculated rates of AKI in agricultural worker research studies may be inaccurate.

In this paper, we compare laboratory serum creatinine values to field-derived Statscan values in order to determine whether the hand-held system can reliably be used to measure serum creatinine under working conditions in a remote tropical location. In addition, we assess the impact of adjusted and unadjusted POC creatinine values on determination of cross-shift AKI and the implications for calculating rates of kidney injury in agricultural worker cohort studies.

## Methods

### Study overview

Two studies were performed in the Escuintla Department on Guatemala’s Pacific coast. The Escuintla region of Guatemala is considered a tropical monsoon climate due to its high average monthly temperatures and typical “wet” and “dry” seasons. Escuintla has an average monthly high of 32° Celsius, and the sugarcane harvest is part of the “dry” season, with less than 10 cm of rain per month, although average humidity is high[27]. The sugarcane harvest season lasts six months from mid-November to early May, and sugarcane cutters are out in the field for 10-hour shifts under hot and humid conditions.

The first study took place during the 2016–2017 harvest and the second study during the 2017–2018 harvest season. Study subjects were recruited from among the sugarcane harvesters working for Pantaleon, an agricultural company operating in Mexico, Guatemala, Honduras, Nicaragua, and Brazil. Participating subjects provided written informed consent. Data from these two studies were used as a convenience sample in this analysis. The studies were approved by the Institutional Review Board (IRB) of the University of Colorado and in Guatemala by the Comite de Etica, Facultad de Medicina, Universidad Francisco Marroquin-Hospital Universitario Esperanza (2016–2017 study) and ZUGUEME Comite Etica Independiente (2017–2018 study).

### Derivation cohort

**Fig 1:**
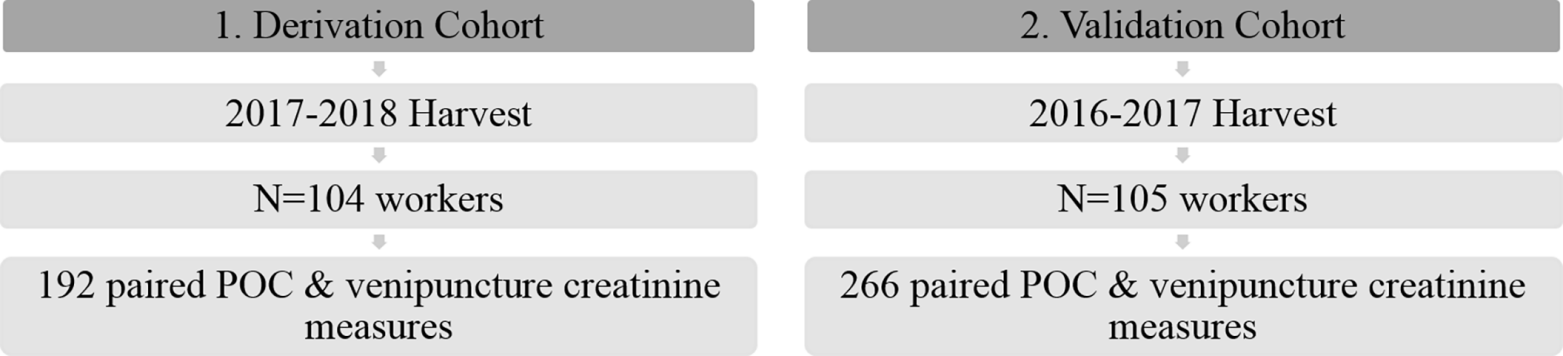
Validation and cohort characteristics.

A random selection of participants was recruited and consented during the 2017–2018 harvest. Study participants were over the age of 18, were cane cutters, and had completed the pre-employment hiring process where their kidney function was measured and they were hired if their estimated glomerular filtration rate (eGFR) was ≥90 ml/min/1.73 m^2^. Data were collected during four days in January 2018. This cohort consisted of 192 paired samples from 104 (Fig 1). Average temperature for data collection days ranged from 22.4–28.7 degrees Celsius.

### Validation cohort

A random selection of participants was recruited and consented during the 2016–2017 harvest, including cane cutters and workers that planted seed (≥18 years old). The pre-employment hiring cut-off for their kidney function for the 2016–2017 harvest was ≥ 60 ml/min/1.73m^2^. The validation cohort consisted of 266 paired blood and finger stick samples from 105 workers at three different time points over the span of three months, from February to April 2017. Average temperature for data collection days ranged from 26.3–35.5 degrees Celsius.

### Creatinine measures

In both cohorts, finger stick samples for Statscan analysis were drawn at the end of a worker’s 8–10 hour shift, with one drop of capillary blood applied to the Statscan sensor according to the manufacturer’s instructions by research personnel trained in the use of the POC device. Venipuncture samples for laboratory analysis were drawn concurrently with finger sticks at the end of the worker’s shift, and sent refrigerated to an independent, licensed clinical laboratory (Herrera Llerandi laboratory, Guatemala City, Guatemala). Creatinine values were performed by the laboratory using the Creatinine Jaffe Generation 2 method, which is a kinetic alkaline picrate method. In the Herrera-Llerandi laboratory, internal controls are run daily, with an external control run once per month. For this reason, blind duplicates were not conducted. Creatinine results higher than 1.4 were re-analyzed to confirm the result.

All Statscan sensors passed precision and linearity testing in the reportable range of 0.3–12.0 at the beginning and end of each study period. Reagent strips were tested at beginning and end of each month, and were discarded and replaced if values were incorrect.

### Rates of AKI

Rates of AKI during a shift were determined by comparing the pre- and post-shift POC creatinine values. Procedures in the field were the same for pre-shift and post-shift POC creatinine collection. POC meters and creatinine strips were stored in coolers during transportation and used under shade while in the field. AKI was defined using the Kidney Disease Improving Global Outcomes (KDIGO) criteria, which is defined as an increase in creatinine of ≥ 0.3 mg/dL or ≥1.5 times the baseline creatinine value. After derivation of a correction factor based on comparison of POC and laboratory creatinine values, we recalculated rates of AKI after applying the correction factor to both the pre- and post-shift values. To determine the impact of the correction factor on AKI rates, we looked at corrected and uncorrected pre- and post-shift creatinine values in the combined derivation and validation cohorts.

### Statistical analysis

To assess the agreement between the Statscan POC creatinine and serum creatinine measures Bland-Altman plots of agreement were first visually assessed[28]. To determine if there was a statistical difference in the measurements of creatinine between Statscan POC and serum paired T-tests were used at a significance level of 0.05. To assess the functional form of the relationship between Statscan POC creatinine and serum creatinine a cubic smoothing spline with 4 degrees of freedom was fit.To determine the necessary adjustment of POC required for agreement with serum creatinine measure, a linear regression model between serum creatinine and Statscan POC creatinine with no intercept was fit. Because of the study design, the independent observation assumption for linear regression was violated. To address this, a linear mixed model with random slope for individual was fit. Variability in individual slopes were assessed. The normality of POC creatinine and serum creatinine measurements were verified using histograms and Q-Q normal plots.

Measures of predictive accuracy were calculated for creatinine values greater than 1.1 mg/dL and 1.3 mg/dL both before and after the correction factor was applied. To temporally validate the correction factor, the correction factor was applied to the 266 paired observations in the validation dataset. Statistical analyses were done using R version 3.4.3[1] and SAS version 9.3 (Cary, NC).

## Results

We initially analyzed the derivation cohort consisting of 104 field workers representing a total of 192 paired end-of-work shift Statscan and laboratory measures of creatinine. The workers were predominantly young males, with a high prevalence of pre-diabetes, though not full Diabetes Mellitus II. Baseline data are given in Table 1. On average, the POC creatinine measured by Statscan was 0.20 (95% CI: -0.24, -0.17) mg/dL higher than the laboratory serum creatinine measure, as shown in Table 2.

**Table 1:** Demographic characteristics of study populations.

Participant Characteristics	Derivation Cohort 2017–2018	Validation Cohort 2016–2017	p-value between cohorts
**Number of participants (N)**	104	105	-
**Age, years, mean (SD)**	29 (7)	30 (9)	0.24
**Male Gender (N, %)**	104 (100%)	105 (100%)	-
**Body Mass Index (kg/m^2^), mean (SD)**	23 (4)	24 (3)	0.77
**Prediabetes (HbA1c: 5.7–6.4%) (N, %)**	13 (13%)	53 (51%)	<0.01
**Job Type**			
**Cane cutter (N, %)**	104 (100%)	83 (79%)	<0.01
**Seeder (N, %)**	0	22 (21%)	
**Kidney function before the start of the harvest season**			
Baseline creatinine* (mg/dL), mean (SD)	0.85 (0.14)	0.90 (0.15)	0.01
Baseline eGFR* (mL/min/1.73 m^2^), mean (SD)	116.8 (13.5)	111.11 (15.60)	<0.01

* Baseline refers to the pre-employment health screening that is conducted at the start of the harvest (August–early November)

**Table 2:** Average creatinine measurements and standard deviations before and after adjustment with the 0.7775 correction factor. In italics are average differences with 95% CI between laboratory measurements and POC values.

	Mean (SD or *95% C*I)	p-value
Serum creatinine	0.88 (0.21)	
Unadjusted POC creatinine	1.08 (0.35)	
*Difference (Serum—POC unadjusted)*	*-0*.*20 (-0*.*24*, *-0*.*17)*	*<0*.*0001*
Adjusted POC creatinine	0.84 (0.27)	
*Difference (Serum—POC adjusted)*	*0*.*04 (0*.*01*, *0*.*07)*	*0*.*004*

**Fig 2:**
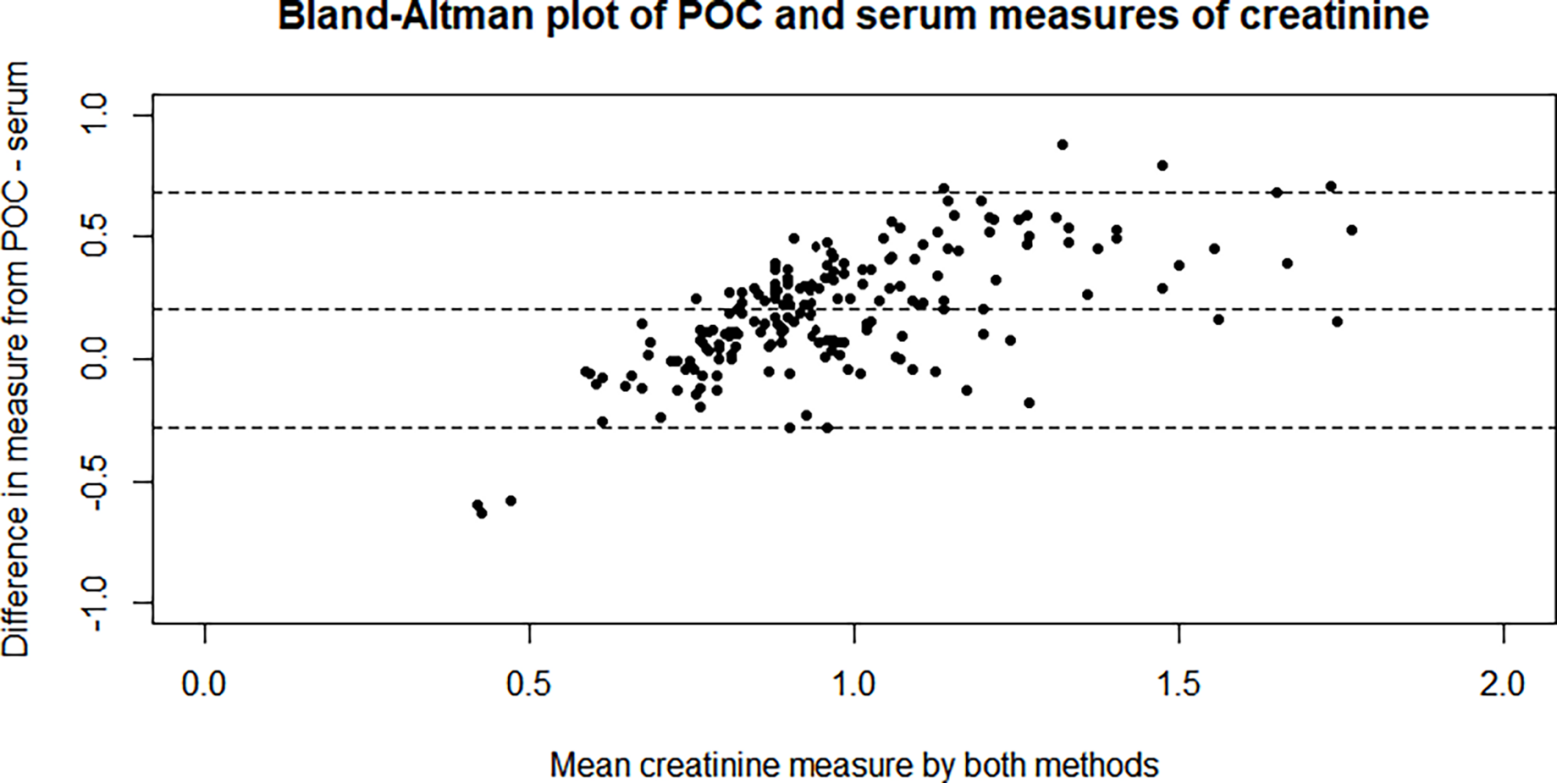
Bland-Altmann plot of agreement between StatScan POC creatinine measurements and serum creatinine measurements. The x-axis represents the mean value of creatinine measured by both methods. The y-axis represents the difference between the POC and laboratory method.

**Fig 3:**
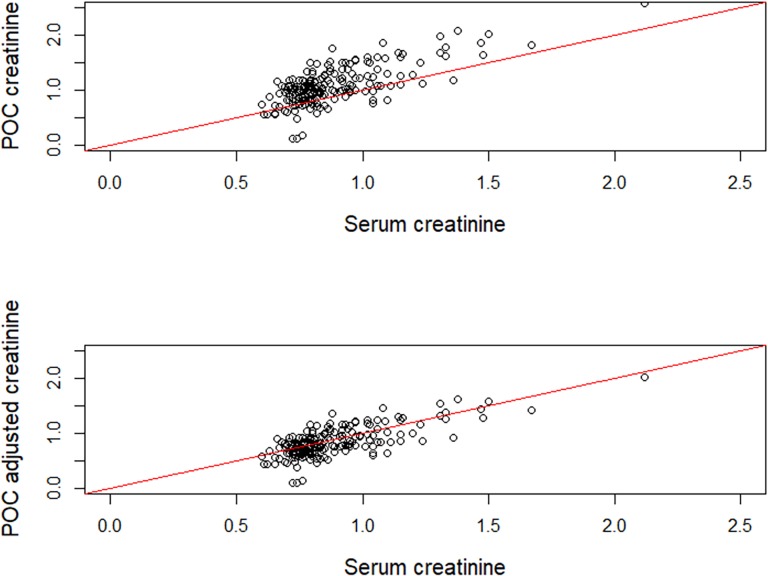
Scatter plot between StatScan POC creatinine measurements and serum creatinine measurements at two time points in 100 individuals. (Upper) Agreeement prior to adjustment. (Lower) Agreement after adjustment. The red line indicates perfect agreement. R Core Team (2017). R: A language and environment for statistical computing. R Foundation for Statistical Computing, Vienna, Austria. URL https://www.R-project.org/.

Visual inspection of the agreement between POC creatinine and serum creatinine measures via a Bland-Altman plot showed proportional bias, the disagreement was greater at higher values of serum creatinine (Fig 2). The POC consistently overestimated the creatinine by an average of 22.3% (95% CI: 19.8%, 24.7%). The adjustment factor (Eq 1) was applied and the agreement between the two measures became much closer aligned (Table 2). A visual representation of the improved agreement is shown in Fig 3.

SerumCreatinine=0.7775*StatscanPOCCreatinine (1)

To address concerns about dependence among repeated measurements in our sample, we re-fit the linear model with no intercept allowing for a random slope for individuals. The resulting model showed that the slopes did not vary among individuals (covariance estimate: 0.0002). Using the random slope model, the estimate for the correction factor changed from 0.7775 to 0.7779. In respect to external validity, we chose the model that treated the observations as independent considering these results.

Performance measures were calculated in the unadjusted and adjusted POC ability to determine creatinine values above 1.1 mg/dL and 1.3 mg/dL (Table 2). For both, levels of sensitivity dropped while specificity increased for the adjusted POC. The adjusted POC saw an increase in positive predictive value with only a marginal decline in negative predictive value. The overall accuracy increased from 0.71 to 0.88 and 0.85 to 0.97 for the 1.1 mg/dL and 1.3 mg/dL cutoffs respectively with the adjustment.

To validate our findings, the correction factor derived from the derivation cohort was applied to the 266 observations collected from 105 workers in the 2016–2017 validation cohort, and the same performance measures were calculated (Table 3). Changes in statistical measures between the unadjusted and adjusted values in the validation cohort were consistent with those seen in the original testing cohort.

**Table 3:** Comparison of performance measures between unadjusted POC creatinine values and adjusted creatinine values in determining creatinine levels above 1.1 mg/dL and 1.3 mg/dL when compared to serum creatinine measures. Classification replicated in a dataset from the previous year to demonstrate external validity.

	Derivation Cohort (2017–2018 Harvest)				Validation Cohort (2016–2017 Harvest)			
	Cutoff 1.1		Cutoff 1.3		Cutoff 1.1		Cutoff 1.3	
	Unadjusted	Adjusted	Unadjusted	Adjusted	Unadjusted	Adjusted	Unadjusted	Adjusted
Sensitivity	0.90	0.70	0.91	0.73	0.96	0.80	0.91	0.78
Specificity	0.69	0.90	0.85	0.99	0.41	0.83	0.65	0.95
Positive Predictive Value	0.25	0.45	0.26	0.80	0.27	0.52	0.20	0.58
Negative Predictive Value	0.98	0.96	0.99	0.98	0.98	0.95	0.99	0.98
Accuracy	0.71	0.88	0.85	0.97	0.51	0.82	0.68	0.93
AUC	0.79	0.80	0.88	0.86	0.68	0.81	0.78	0.87

### Application of POC data correction in assessment of AKI

To illustrate the potential implications of POC data correction in the field, we examined corrected and uncorrected values to a separate set of paired, pre- and post-shift POC creatinine measures obtained in both cohorts of workers. When using unadjusted values with the validation cohort, there were 202 (72%) AKI events (increase in creatinine of 0.3 mg/dL or greater or a 1.5-fold rise in creatinine from pre-shift). When adjusted creatinine values were used, there were 159 (57%) AKI events (Table 4).

**Table 4:** Impact of application of correction factor on rates of AKI in the derivation and validation cohorts.

**Derivation Cohort, 2017–2018 Harvest (N = 104)**	**Day 1 (n = 96)**	**Day 6 (n = 96)**	**Total (n = 192)**	
Unadjusted AKI	78 (81%)	44 (46%)	122 (64%)	
Adjusted AKI	75 (78%)	41 (43%)	116 (60%)*	
**Validation Cohort, 2016–2017 Harvest (N = 105)**	**February (n = 102)**	**March (n = 87)**	**April (n = 92)**	**Total (n = 281)**
Unadjusted AKI	77 (75%)	60 (69%)	65 (71%)	202 (72%)
Adjusted AKI	64 (63%)	42 (48%)	53 (58%)	159 (57%)*

*Chi-sq test comparison between adjusted AKI rates between Derivation cohort and Validation cohort, p-value = 0.62.

## Discussion

Tropical field measurements using a POC instrument overestimate agricultural workers’ creatinine levels, consequently exaggerating the rate of AKI that occurs across the work shift. The use of a statistical adjustment improves positive predictive values and overall accuracy. It is important for kidney health researchers and workplace screening programs to ensure that POC results have been validated with side-by-side laboratory serum creatinine values, or, at least, apply a validated correction factor. Application of an adjustment factor of 0.7775, which we were we able to derive and validate in a population of sugarcane workers in Guatemala, leads to better specificity and a better positive predictive value, with an improvement in accuracy to > 0.9 (Table 3). This allows for more accurate detection of AKI and CKD in remote field locations.

Importantly, our data suggest that this correction results in a significant difference in the reported incidence of AKI in agricultural workers. Recent studies in agricultural settings have reported rates of AKI using StatScan or a similar device from another manufacturer [12, 14, 29, 30], with AKI reported AKI rates ranging from 12–33%. We would expect that other devices would produce similar discrepancies, and further research should be conducted to confirm the need for an adjustment for those devices. From a research standpoint, it is important to make accurate case determinations of AKI, especially in studies that intend to identify meteorological, environmental, and other risk factors, as well as in studies with interventions intended to mitigate such risks.

As with AKI, accurate creatinine measures are vitally important both for research into the etiology of CKD. Our analysis shows that the POC Statscan system overestimated creatinine by 22%. Of note, use of this device as a CKD screening tool in Rivas, Nicaragua, which has a similar climate, showed an average creatinine of 0.72 mg/dL with laboratory testing and 1.04 mg/dL with the POC device, giving a slightly larger discordance than the one reported in our study[9]. This suggests that rates of CKD may be overestimated. For instance, a seemingly elevated POC creatinine value of 1.54 mg/dL might in fact be below the upper limit of normal (1.2 mg/dL) if checked using standard laboratory measures. Because creatinine is a key component in the calculation of estimated glomerular filtration rate (eGFR)[31], and is an important part of the definition of CKD, the same cautions apply to research and intervention efforts that address the similar epidemics that have now been described in Latin America, Egypt, Sri Lanka, India, and Cameroon[32, 33]. An accurate assignment of eGFR is essential to understanding the prevalence, incidence, response to interventions such as water, rest, and shade[34], and contributing etiologic factors, as well as natural history and clinical course of CKD.

From a workforce hiring perspective, if creatinine is used as a screening tool, as is customary in much of the sugar cane industry[35], a 22% overestimate of creatinine could harm a worker’s chance of gaining seasonal employment or result in them being given a less desirable job. In order to avoid unfairly denying employment or making unnecessary workplace accommodations, we have recommended that employer clinics recheck high POC measurements and confirm POC creatinine values that fall above the normal range, before making a hiring or job placement decision. Agricultural companies also have a vested interest in maintaining a healthy workforce. In remote areas where laboratory testing is impractical, accurate POC determinations of AKI are necessary in order to implement appropriate prevention and treatment strategies.

Both AKI and CKD often occur in remote, rural locations without ready access to laboratory services. Handheld POC devices, including the Statscan system, are routinely utilized due to their ease-of-use, portability, and speed. In our experience, they provide important, real-time information for workers and for occupational health care providers. Over the past two years, we have used POC devices at time of pre-employment, mid-season testing, and end of season testing in extreme, remote work conditions of high heat, humidity, and wind. Thus, we continue to be enthusiastic about the feasibility of their use, with the caveat that all results are corrected and periodically compared to a laboratory gold standard.

Our results were generated using two different sets of paired specimens from two groups of workers, over two consecutive seasons. As such, we have confidence in the reliability of our findings. The environmental conditions found in Guatemalan sugar cane fields are similar to the conditions experienced by many agricultural workers in tropical regions across the globe. That being said, we caution that the correction factor may not be applicable in all settings. We only tested specimens under a narrow range of temperature and humidity. Further studies are needed to determine if this formula is generalizable to other field environments, including wider ranges of temperature and humidity. As mentioned above, statistical correction may vary for other brands of POC instruments on the market. We were unable to determine in this study why the StatScan results were more abnormal in the field environment than in previous hospital environments. Another limitation in this analysis is that we were not able to collect pre-shift blood creatinine values in order to ensure values in the morning had the same sampling difference between POC and blood creatinine compared to discrepancy seen with the post-shift values. As a final limitation, it is possible, although unlikely, that the serum creatinine measurements that we used for benchmarking the POC results are inaccurate. We consider this extremely unlikely, given that those tests were conducted independently, and blinded by a certified, approved clinical laboratory, using a well-established, well-standardized clinical laboratory method which is accredited by ISO standard 15189:2012, extended by the Guatemalan Accreditation Office under the supervision of the Ministry of Economy and is extended by the Ministry of Public Health & Social Assistance of Guatemala.

## Conclusion

The Statscan meter has many important benefits, including portability, low cost, convenience, and immediacy of results. While it is not as accurate as laboratory testing in tropical field conditions, this limitation can be overcome with an adjustment factor. Critical consideration of the validity of POC results is warranted when drawing conclusions regarding the prevalence, incidence, progression, and etiology of AKI and CKD in field research and agricultural worker health management.
